# T CD3^+^CD8^+^ Lymphocytes Are More Susceptible for Apoptosis in the First Trimester of Normal Human Pregnancy

**DOI:** 10.1155/2014/670524

**Published:** 2014-07-20

**Authors:** Dorota Darmochwal-Kolarz, Ewelina Sobczak, Piotr Pozarowski, Bogdan Kolarz, Jacek Rolinski, Jan Oleszczuk

**Affiliations:** ^1^Department of Obstetrics and Perinatology, Medical University of Lublin, Ulica Jaczewskiego 8, 20-950 Lublin, Poland; ^2^Department of Clinical Immunology, Medical University of Lublin, 20-950 Lublin, Poland

## Abstract

*Aims.* Normal human pregnancy is a complex process of many immunoregulatory mechanisms which protect fetus from the activation of the maternal immune system. The aim of the study was to investigate the apoptosis of lymphocytes in peripheral blood of normal pregnant patients and healthy nonpregnant women. *Methods.* Sixty pregnant women and 17 nonpregnant women were included in the study. Lymphocytes were isolated and labeled with anti-CD3, anti-CD4, and anti-CD8 monoclonal antibodies. Apoptosis was detected by CMXRos staining and analyzed using the flow cytometric method. *Results.* We found significantly higher apoptosis of total lymphocytes in peripheral blood of pregnant patients when compared to healthy nonpregnant women. The percentage of apoptotic T CD3^+^CD8^+^ cells in the first trimester was significantly higher when compared to the third trimester of normal pregnancy. The ratio of T CD3^+^CD4^+^ : T CD3^+^CD8^+^ apoptotic lymphocytes was significantly lower in the first trimester when compared to other trimesters of pregnancy and to both of the phases of the menstrual cycle. *Conclusions.* The higher apoptosis of T CD3^+^CD8^+^ lymphocytes and the lower ratio of T CD3^+^CD4^+^ : T CD3^+^CD8^+^ apoptotic cells in the first trimester of normal pregnancy may suggest a higher susceptibility of T CD3^+^CD8^+^ cells for apoptosis as a protective mechanism at the early stage of pregnancy.

## 1. Introduction

Programmed cell death is an important immunoregulatory mechanism that occurs under a wide variety of physiological and pathological situations. It plays a significant role in the maintenance of homeostasis in almost all animal tissues including the immune system [1–4]. Apoptosis consists of distinct biochemical and sequential morphological changes. This includes condensation and segmentation of both chromatin and cytoplasm and extensive fragmentation of chromosomal DNA into nucleosome units forming apoptotic bodies which contain dense masses of chromatin and intact organelles and cellular fragmentation [1–4]. Two major apoptotic pathways have been described in mammals: the extrinsic death-receptor signaling pathway and the intrinsic mitochondrial pathway, resulting in caspase cascade activation [1–4]. Apoptosis is an efficient mechanism for eliminating activated lymphocytes by a Fas/FasL-mediated programmed cell death process in the immune system. This process is called activation induced cell death (AICD) [5, 6]. The programmed cell death mediated by Fas/FasL is mainly a part of the regulation of an immune response and homeostasis of body tissues. Many studies suggest additional functions of this system, especially an important role in the pathogenesis of many diseases characterized by decreased or excessive apoptosis [5, 6].

It is believed that programmed cell death is considerable during embryogenesis, carcinogenesis, and host defense [1–4]. Several findings have suggested that apoptosis plays an important role in the normal development, remodeling, and aging of the placenta [7–9]. Moreover, it has been demonstrated that apoptosis increases as pregnancy progresses suggesting that it is a normal physiological phenomenon throughout gestation [7].

There are studies which revealed that apoptosis present in peripheral blood is involved in the process of destroying fetal DNA fragments. Kolialexi et al. showed that the increased trafficking of fetal cells in maternal peripheral blood stimulates apoptosis, possibly to prevent maternal rejection of fetus [10]. On the other hand, during normal pregnancy, neutrophil apoptosis is delayed, which can explain neutrophilia in pregnancy [11].

The purpose of our study was to estimate the populations of total apoptotic lymphocytes as well as T CD3^+^, T CD4^+^, and T CD8^+^ apoptotic lymphocytes in peripheral blood of pregnant women in the first, second, and third trimesters of physiological pregnancy and healthy nonpregnant women in both of the phases of the menstrual cycle.

## 2. Material and Methods

The patients participating in the study were admitted to the Department of Obstetrics and Perinatology of the Medical University of Lublin. The study population included 60 uncomplicated pregnant women within the age range of 21–41. They were divided into three groups—20 patients were in the first trimester, 20 in the second trimester, and 20 in the third trimester of normal pregnancy. All pregnancies were singleton. Complete medical, surgical, and social history was obtained from all women. The control group comprised 17 nonpregnant healthy women who did not take the contraceptive pills. They had regular biphasic (ovulatory) cycles. Blood samples from nonpregnant women were collected twice, between 6 and 9 and 19 and 22 days of the menstrual cycle. The menstrual cycles of women were monitored. The preovulatory LH surge, the rise in the basal body temperature (BBT), and the changes in cervical mucus were taken into account to assess the menstrual cycle. The study design was accepted by the local ethics committee. Informed consent from the patients for peripheral blood sampling was obtained.

Twenty milliliters of peripheral blood was taken from each patient by venipuncture in sterile conditions and collected in heparinized tubes. Blood samples were diluted 1 : 1 in 20 mL physiological buffered saline (PBS) (Serum and Vaccine Factory, Biomed, Lublin, Poland). Peripheral blood mononuclear cells (PBMC) were separated on the lymphocyte separation medium: Gradisol L (Aqua Medica, Lodz, Poland). They were centrifuged for 20 min at 700 ×g, collected from the interface with a Pasteur pipette, and washed twice in 2 mL of PBS by centrifugation for 5 min at 700 ×g. Twenty-five *μ*L of isolated cells was mixed with 475 *μ*L Turk's solution (10% acetic acid), and the total number of cells was determined using the microscope. We have used 0.4% Trypan Blue solution (Sigma, Germany) to rate the lifeless cells using the microscope.

CMXRos (chloromethyl-X-rosamine) stored at −20°C (following the manufacturer's instructions) was dissolved in 94 *μ*L of dimethylsulfoxide (DMSO) (Sigma, USA) to give stock solution. Next, we diluted stock solution mixing 10 *μ*L of stock solution with 190 *μ*L of DMSO. It could be stored for a few months in 4°C. The suspension of isolated cells (2.5 *μ*L–500000 of cells) was put in 0.5 mL of culture medium (10% FCS and RPMI). Cells were incubated with 5 *μ*L of CMXRos in the growth medium at 37°C and 5% CO_2_. After 15 minutes of the incubation, the cells were labeled by direct staining with 2.5 *μ*L of monoclonal antibodies (BD Biosciences, San Jose, California, USA) in the following combinations:

1. anti-CD3 (FITC) and anti-CD4 (PE),
2. anti-CD3 (FITC) and anti-CD8 (PE),
3. anti-glycophorin A (FITC).

The cells were incubated with antibodies and CMXRos for the next 15 minutes. The anti-glycophorin A antibody (Daco, Denmark) was used to eliminate erythrocytes.

**Figure 1:**
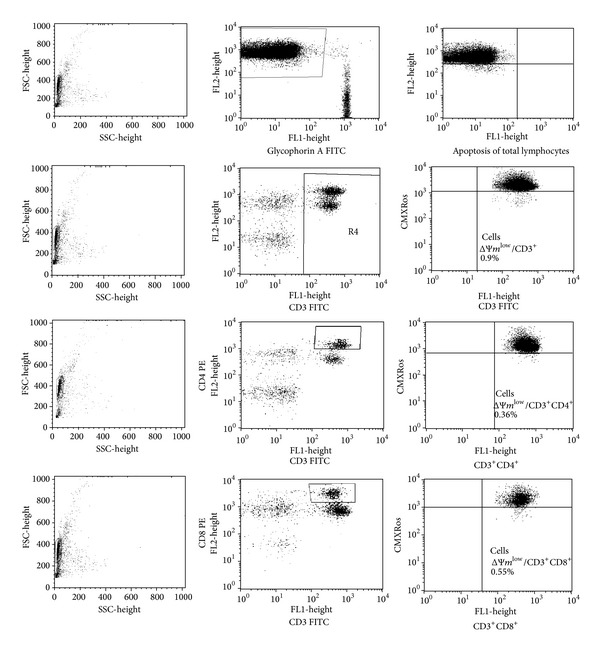
The strategy of gating and analyzing of total apoptotic lymphocytes and T CD3^+^, T CD3^+^CD4^+^, and T CD3^+^CD8^+^ apoptotic lymphocytes.

*Flow Cytometric Analysis Strategy of Gating and Analyzing of Apoptotic Cells.* The cells were collected using FACSCalibur flow cytometer equipped with 488 nm argon laser (Becton Dickinson) and analyzed with CellQuest Software. Lymphocytes were classified according to forward scatter (FSC) and side scatter (SSC). The cells were gated in FSC/SSC dot blots. In the case of assessment of total lymphocyte apoptosis, erythrocytes (glycophorin A^+^ events) were gated out and disruption of mitochondrial potential was calculated as shown on histograms or dot plots. The apoptosis results represent the percentage of apoptotic events within total lymphocytes. Similarly, apoptosis of T CD3^+^, T CD3^+^CD4^+^, and T CD3^+^CD8^+^ lymphocytes was calculated. Firstly, the regions for T CD3^+^, T CD3^+^CD4^+^, and T CD3^+^CD8^+^ lymphocytes were found. Next, the percentages of T CD3^+^, T CD3^+^CD4^+^, and T CD3^+^CD8^+^ lymphocyte apoptosis were estimated using CMXRos. The apoptosis results represent the percentage of apoptotic events within T CD3^+^, T CD3^+^CD4^+^, and T CD3^+^CD8^+^ lymphocytes. The strategy of gating and analyzing of total apoptotic lymphocytes and T CD3^+^, T CD3^+^CD4^+^, and T CD3^+^CD8^+^ apoptotic lymphocytes was shown in Figure 1.

The results were presented as median with the interquartile ranges. Statistical differences between groups were estimated using standard Mann-Whitney *U* test and Wilcoxon test. In the cases of the comparison of the follicular and luteal phases of menstrual cycle a standard Wilcoxon test was used. Differences at the *P* < 0.05 level were considered as statistically significant. Statistica 7.1 PL software was applied for statistical analysis.

## 3. Results

The percentage of total apoptotic lymphocytes was significantly higher in total pregnant women (in the first, second, and third trimesters) when compared to the follicular phase of nonpregnant women (1.57% versus 1.35%; *P* < 0.05). In the first trimester of normal pregnancy the percentage of apoptotic lymphocytes was significantly higher in comparison with both the follicular (1.78% versus 1.35%; *P* < 0.05) and the luteal phases of the menstrual cycle (1.78% versus 1.52%; *P* < 0.05). The percentage of total apoptotic lymphocytes was higher in the first trimester than in the second and third trimesters of normal pregnancy but the differences were not statistically significant (I trimester versus II trimester: 1.78% versus 1.39%, NS; I trimester versus III trimester: 1.78% versus 1.43%, NS). There were no differences between the percentages of apoptotic lymphocytes in the second and third trimesters of pregnancy (II trimester versus III trimester: 1.39% versus 1.43%, NS). The percentage of apoptotic lymphocytes was higher in the luteal than in the follicular phase of the menstrual cycle but the differences were not statistically significant (1.52% versus 1.35%, NS).

The percentage of apoptotic T CD3^+^ lymphocytes was significantly lower in the third trimester of normal pregnancy in comparison with the follicular (1.02% versus 1.11%; *P* < 0.05) and luteal phases of the menstrual cycle (1.02% versus 1.25%; *P* < 0.05). The percentage of apoptotic T CD3^+^ lymphocytes was higher in the first trimester than in the second and third trimesters of pregnancy and in the phases of the menstrual cycle but the differences were not statistically significant (I trimester versus II trimester: 1.20% versus 1.04%, NS; I trimester versus III trimester: 1.20% versus 1.02%, NS; I trimester versus follicular phase: 1.20% versus 1.11%, NS; I trimester versus luteal phase: 1.20% versus 1.25%, NS). There were no differences between second and third trimesters of pregnancy (1.04% versus median: 1.02%, NS).

The percentage of apoptotic T CD3^+^CD4^+^ cells of nonpregnant women was significantly higher than that of pregnant women (0.45% versus 0.41%; *P* < 0.05). Furthermore, the percentage of apoptotic T CD3^+^CD4^+^ cells in the luteal phase of the menstrual cycle was significantly higher than that of pregnant women (0.50% versus 0.41%; *P* < 0.05). The percentage of apoptotic T CD3^+^CD4^+^ cells was significantly lower in the first and the third trimesters of normal pregnancy when compared to the luteal phase of the menstrual cycle (I trimester versus luteal phase: 0.39% versus 0.50%; *P* < 0.05, and III trimester versus luteal phase: 0.42% versus 0.50%; *P* < 0.05). There were no differences between the first, second, and third trimesters of normal pregnancy (I trimester versus II trimester: 0.39% versus median: 0.44%, NS; I trimester versus III trimester: 0.39% versus 0.42%, NS; II trimester versus III trimester: 0.44% versus 0.42%, NS).

**Figure 2:**
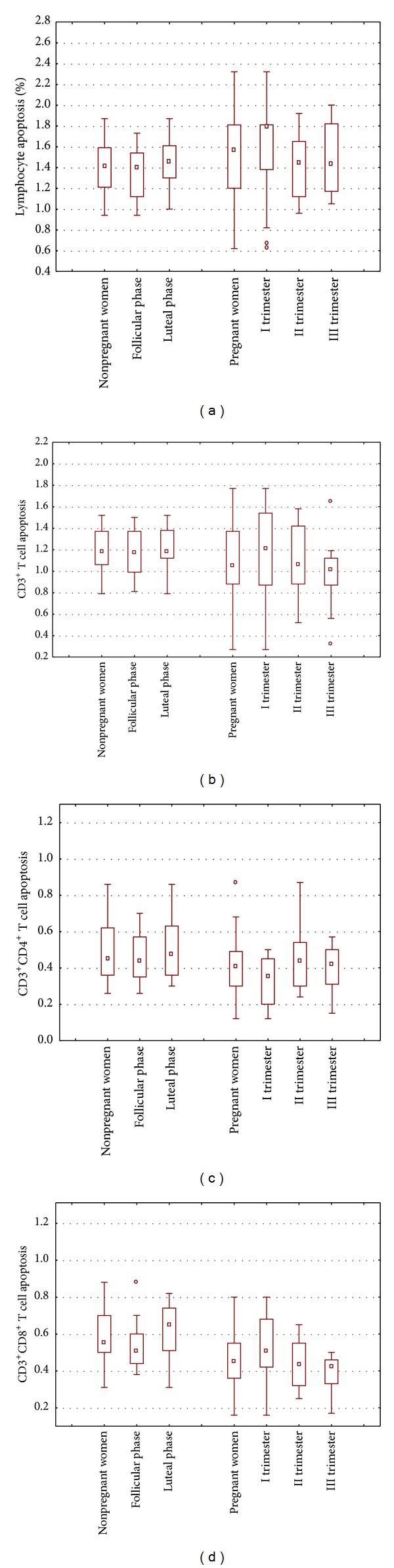
The percentages of total lymphocytes and T CD3^+^, T CD3^+^CD4^+^, and T CD3^+^CD8^+^ apoptotic lymphocytes; lymphocyte apoptosis in peripheral blood of women in the first (*n* = 20), second (*n* = 20), and third trimesters (*n* = 20) of normal pregnancy and in peripheral blood of healthy nonpregnant women in the phases of the menstrual cycle (*n* = 17).

The percentage of apoptotic T CD3^+^CD8^+^ lymphocytes in normal pregnant patients was significantly lower in comparison with nonpregnant women (0.45% versus 0.55%; *P* < 0.001). The percentage of T CD3^+^CD8^+^ apoptotic lymphocytes of normal pregnant women was significantly lower when compared to both the luteal and follicular phases of the menstrual cycle (normal pregnancy versus luteal phase: 0.45% versus 0.64%, *P* < 0.05; normal pregnancy versus follicular phase: 0.45% versus 0.51%, *P* < 0.001). The percentage of T CD3^+^CD8^+^ apoptotic cells in the luteal phase was significantly higher than that in the second (luteal phase versus II trimester: 0.64% versus median: 0.44%; *P* < 0.001) and the third trimesters of normal pregnancy (luteal phase versus III trimester: 0.64% versus 0.42%; *P* < 0.001). The percentage of apoptotic T CD3^+^CD8^+^ lymphocytes in the follicular phase of the menstrual cycle was significantly higher when compared to the third trimester of normal pregnancy (0.51% versus 0.42%; *P* < 0.001). Apoptosis of T CD3^+^CD8^+^ lymphocytes was significantly lower in the third trimester when compared to the first trimester of normal pregnancy (0.42% versus 0.54%; *P* < 0.05). There were no differences between the second and third trimesters of normal pregnancy (0.44% versus 0.42%, NS). The percentage values of total apoptotic lymphocytes and T CD3^+^, T CD3^+^CD4^+^, and T CD3^+^CD8^+^ apoptotic lymphocytes are presented in Figure 2.

**Figure 3:**
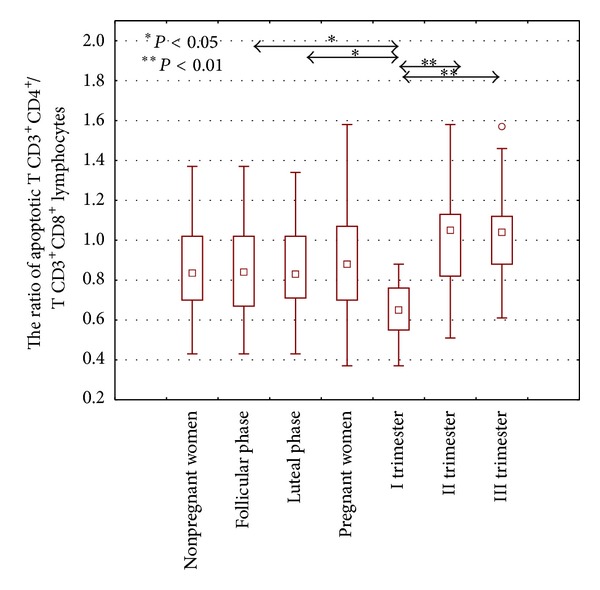
The ratio of T CD3^+^CD4^+^ : T CD3^+^CD8^+^ apoptotic lymphocytes in peripheral blood of women in the first (*n* = 20), second (*n* = 20), and third trimesters (*n* = 20) of normal pregnancy and in peripheral blood of healthy nonpregnant women in the phases of the menstrual cycle (*n* = 17).

The ratio of apoptotic T CD3^+^CD4^+^: T CD3^+^CD8^+^ lymphocytes in the first trimester was significantly lower when compared to the second (0.65 versus 1.05; *P* < 0.001) and to the third trimesters of normal pregnancy (0.65 versus 1.04; *P* < 0.001). Moreover, the ratio of T CD3^+^CD4^+^: T CD3^+^CD8^+^ apoptotic lymphocytes in the first trimester of normal pregnancy was significantly lower in comparison with the luteal (0.65 versus 0.83; *P* < 0.05) and follicular phases of the menstrual cycle (0.65 versus 0.84; *P* < 0.05). There were no differences between the second and third trimesters of pregnancy (1.05 versus 1.04, NS). The results of the ratio of T CD3^+^CD4^+^: T CD3^+^CD8^+^ apoptotic lymphocytes are presented in Figure 3. The absolute numbers of total apoptotic lymphocytes and apoptotic T CD3^+^, T CD3^+^CD4^+^, and T CD3^+^CD8^+^ cells in normal pregnant women and in the follicular and luteal phases of the menstrual cycle of nonpregnant women are presented in Table 1.

## 4. Discussion

Apoptosis occurs during normal development and it is important for the right balance between the loss of old, nonfunctional cells and the formation of new cells in the different organs and tissues. Recent findings suggest that Fas and FasL antigens or TRAIL-R-TRAIL are essential for the process of cell death and that any dysfunction in this system can lead to a breakdown in peripheral tolerance [5, 6].

The majority of studies demonstrate apoptosis in the placenta. There are only few documented data describing this process in peripheral blood in physiological pregnancies [7–10, 12].

It is well known that fetal antigens influence the maternal immune system. Apoptotic bodies that are present in peripheral blood induce upregulation of T CD4^+^ and T CD8^+^ cells. Moreover, in the first trimester of pregnancy apoptotic extravillous trophoblast cells are probably the antigenic source. In the second trimester, during the further invasion of trophoblast, uncovered apoptotic syncytiotrophoblast cells enter the maternal circulation. They are briefly opsonized and presented by peripheral antigen presenting cells (APC) [12, 13]. Thus, the tolerance is locally initiated within decidua and maintained until term by more “systemic” antigen presentation. Furthermore, binding CTLA-4 on T cells to B7 costimulatory receptor will deliver an inhibitory signal to T cells [14, 15]. It was observed that activated T cells capable of inducing extravillous trophoblast apoptosis may be inhibited by the CTLA-4 dependent mechanism. In addition to this, it was said that maternal immune cells and their cytokines regulate trophoblast apoptosis [16, 17].

There are many immunomodulatory pathways which can regulate the process of lymphocyte activity in pregnancy. It had been demonstrated that placental exosomes are critical in modulating T cell activation and suppressing effector T cells by enhancing T lymphocyte apoptosis and CD3-zeta loss. In the study of Sabapatha et al. placental exosomes isolated from maternal peripheral circulation suppressed T cell expression of CD3-zeta and JAK3, while inducing SOCS-2. The level of CD3-zeta on T CD8^+^ cells was inhibited 1.43-fold more than in T CD4^+^ cells [18]. Furthermore, an undefined factor capable of downregulating T cell activity has recently been reported as being produced by short-term cultures of placental fragments. The factor was produced by chorionic villi from term placenta. This low molecular weight, heat stable factor was capable of inhibiting the IL-2 dependent proliferation of mouse CTLL-2 cells. The immunosuppressive effect of the factor, which probably can be prostaglandin PGE2, was exerted via the EP4 receptor [19]. In another study, the anergy of T lymphocytes induced by a low molecular weight material present in a human placental supernatant was linked to the defective phosphorylation of TCR CD3 chain [20]. In addition, regeneration and tolerance factor (RTF) expressed in the human placenta induces the production of IL-10 and can regulate immune response in the maternal-fetal relationship [21]. Furthermore, it has been revealed that RTF is expressed during T lymphocyte activation and plays a role in T lymphocyte apoptosis [22, 23].

Other factors which can influence apoptosis of T lymphocytes in pregnancy may be nonclassic HLA-G antigens expressed at the extravillous cytotrophoblast as well as soluble isoforms of HLA-G proteins. Both membrane-bound HLA-G and soluble isoforms of the proteins derived from HLA-G class Ib gene are produced by placental trophoblast cells. The nonpolymorphic soluble HLA-G1 (sHLA-G1) isoform has been reported to be secreted by trophoblast cells at the maternal-fetal interface, suggesting that it may act as immunomodulator during pregnancy. It has been reported that affinity-purified beta2-microglobulin-associated sHLA-G1 enhanced CD95 ligand expression and apoptosis in activated T CD8^+^ cells [24, 25]. Studies performed in mice models revealed that exposure of maternal T cells to H-Y fetal antigen resulted in deletion of 50% of H-Y specific maternal T cells. The remaining H-Y specific T cells were hyporesponsive to H-Y as assayed by decreased proliferative ability and CTL activity. Further experiments revealed that the expression of FasL by fetus, but not by mother, is necessary and sufficient for both components of maternal tolerance to fetal antigens. The authors suggest that Fas interaction with fetal FasL is critical for both deletion and hyporesponsiveness of H-Y reactive T CD8^+^ cells during pregnancy [26].

Some studies indicate that apoptosis in the 16th–19th weeks of pregnancy was higher than that observed in nonpregnant women. The increased trafficking of fetal cells in maternal peripheral blood can stimulate apoptosis, possibly to prevent maternal rejection of the fetus [10]. Moreover, it was revealed that apoptosis rate was further delayed in a linear manner with increasing gestational age [10, 11]. Trophoblast apoptosis in the first trimester may be necessary to further established maternal tolerance to fetus by induction of T cells [27].

It is known that cytotoxic effect of T cells is largely reduced in pregnant women. This can be caused by anergy-inducing mechanisms and/or the increased apoptosis of T cytotoxic lymphocytes [27, 28].

Our results showed that apoptosis of peripheral blood lymphocytes was higher in normal pregnant patients when compared to healthy nonpregnant women. On the other hand, we noticed the lower percentage of T CD3^+^ apoptotic lymphocytes in the third trimester of physiological pregnancy when compared to the phases of the menstrual cycle. Our data also showed significantly higher percentages of T CD3^+^CD8^+^ lymphocyte apoptosis in peripheral blood in the first trimester in comparison with the third trimester of pregnancy. Furthermore, the ratio of T CD3^+^CD4^+^: T CD3^+^CD8^+^ apoptotic lymphocytes was significantly lower in the first trimester when compared to the other trimesters of pregnancy and to the both phases of the menstrual cycle. It can suggest that T CD3^+^CD8^+^ lymphocytes easily go under apoptosis in the first trimester of physiological pregnancy. Probably, it is because of the fact that the fetus must be more protected at the beginning of the pregnancy. Furthermore, increased apoptosis of peripheral blood lymphocytes in the first trimester of pregnancy may be associated with the increased implantation related apoptosis.

The increased expressions of APO-1/Fas antigen on T lymphocytes and NK cells in the luteal phase of the menstrual cycle might reflect aspects of the phenomenon of preparation for blastocyst implantation [29]. The lower rate of T cell apoptosis could correlate with the downregulation of APO-1/Fas antigen. Moreover, the reduced expression of APO-1/Fas antigen (CD95) on lymphoid cells in peripheral blood can lead to the dysfunction of apoptosis in pregnant women. Reinhard et al. observed that the expression of APO-1/Fas antigen on T cells was significantly elevated during pregnancy and in the postdelivery phase, whereas Fas-mediated apoptosis of peripheral blood T lymphocytes of pregnant patients was significantly reduced when compared to nonpregnant women [30]. Similarly, we observed the lower percentage of peripheral blood T CD3^+^ apoptotic lymphocytes in the normal third trimester pregnant patients when compared to nonpregnant women.

There are many factors that can influence apoptosis of T lymphocytes during pregnancy. Steroids hormones were taken under consideration [30–33]. Shirshev et al. studied the effects of chorionic gonadotropin (CG), estradiol, progesterone, and their physiological combinations on apoptosis of human peripheral blood T lymphocytes [31]. The reproductive hormones effectively regulated apoptosis of peripheral blood T lymphocytes. The hormonal combinations were shown to display differential effects on different T cell subpopulations. The hormonal combinations specific for the first trimester of pregnancy stimulated apoptosis of T CD8^+^ lymphocytes and inhibited apoptosis of T CD4^+^ cells [23]. The results of our study are consistent with their observations. We observed the lower ratio of T CD3^+^CD4^+^ : T CD3^+^CD8^+^ apoptotic lymphocytes in the first trimester when compared to the second and third trimesters of normal pregnancy. It suggests that the apoptosis rate of T CD3^+^CD8^+^ lymphocytes is higher than T CD3^+^CD4^+^ lymphocytes in the first trimester of normal pregnancy in comparison with other trimesters.

Our findings showed that the percentages of apoptotic T CD3^+^CD4^+^ in pregnant patients were significantly lower than those in nonpregnant women. It seems that hormones that are released during pregnancy could inhibit apoptosis, especially apoptosis of T CD3^+^CD4^+^ lymphocytes [30]. There are some studies that revealed that menstrual steroid hormones have multiple effects on the function of immune cells [30–33]. The steroid hormones are probably the most important factors regulating the mechanism of apoptosis [30–33]. Hofmann-Lehmann et al. studied the rate of apoptosis in peripheral blood lymphocytes in female and male cats. They observed lower rates of apoptosis in peripheral blood lymphocytes of female cats than those of male cats. They noticed the correlation of apoptosis rate with 17 beta-estradiol concentrations but not with progesterone blood levels. The authors concluded that 17 beta-estradiol in physiological concentrations may protect peripheral lymphocytes from apoptosis [32].

The increased rate of T CD3^+^CD8^+^ lymphocyte apoptosis in the first trimester of normal pregnancy can suggest that alloreactive T CD3^+^CD8^+^ lymphocytes are removed. It seems to be one of the mechanisms of protecting fetus from the maternal rejection.

The higher percentage of T CD3^+^CD8^+^ apoptotic lymphocytes and the lower ratio of T CD3^+^CD4^+^ : T CD3^+^CD8^+^ apoptotic cells in the first trimester of normal pregnancy may suggest a higher susceptibility of T CD3^+^CD8^+^ cells for apoptosis as a protective mechanism at the early stage of pregnancy. Furthermore, increased apoptosis of peripheral blood lymphocytes in the first trimester of pregnancy may be associated with the increased implantation related apoptosis.

## Figures and Tables

**Table 1:** The absolute numbers of total apoptotic lymphocytes and apoptotic T CD3^+^, T CD3^+^CD4^+^, and T CD3^+^CD8^+^cells (cells/mm^3^ of blood) in peripheral blood of healthy pregnant women and healthy nonpregnant women.

	Nonpregnant women (*n* = 34)	Follicular phase (*n* = 17)	Luteal phase (*n* = 17)	Pregnant women (*n* = 60)	First trimester (*n* = 20)	Second trimester (*n* = 20)	Third trimester (*n* = 20)
Lymphocytes apoptosis median (interquartile ranges)	27.18 (23.19–29.30)	24.11 (22.49–27.36)∗	27.26 (25.74–29.30)	28.03 (24.0–31.34)	29.59 (26.01–31.34)∗	27.91 (22.46–31.25)	27.50 (24.39–31.28)

T CD3^+^ cell apoptosis median (interquartile ranges)	22.83 (19.97–24.57)∗	21.55 (19.97–22.87)	24.39 (20.57–24.57)∗∗	19.39 (14.85–24.39)∗	23.55 (14.10–27.32)	19.26 (13.60–22.71)	18.98 (17.04–22.71)∗∗

T CD4^+^ apoptosis median (interquartile ranges)	8.00 (6.40–11.13)∗∗	7.87 (6.84–9.95)∗∗	8.51 (6.41–11.28)∗∗∗	7.08 (4.98–9.08)∗∗	5.83 (4.24–8.86)^∗,∗∗,∗∗∗^	8.05 (5.83–9.66)∗	6.93 (6.04–8.26)

T CD8^+^ apoptosis median (interquartile ranges)	10.50 (8.54–13.13)∗∗∗∗	9.58 (8.32–11.0)^∗,∗∗∗,∗∗∗∗^	12.01 (10.15–10.15)^∗,∗∗∗∗,∗∗∗∗^	7.78 (6.59–10.51)∗∗∗∗	9.29 (6.81–12.75)	8.02 (6.82–10.11)∗∗∗∗	6.95 (6.40–8.72)^∗∗∗,∗∗∗∗^

*P < 0.05; ∗∗*P* < 0.01; ∗∗∗*P* < 0.005; ∗∗∗∗*P* < 0.001.

## References

[1] Wyllie AH, Kerr JFR, Currie AR (1980). Cell death: the significance of apoptosis. *International Review of Cytology*.

[2] Raff MC (1992). Social controls on cell survival and cell death. *Nature*.

[3] Wyllie AH (1994). Death gets a break. *Nature*.

[4] Ross DW (1997). Apoptosis. *Archives of Pathology and Laboratory Medicine*.

[5] Lynch DH, Ramsdell F, Alderson MR (1995). Fas and FasL in the homeostatic regulation of immune responses. *Immunology Today*.

[6] Nagata S, Golstein P (1995). The Fas death factor. *Science*.

[7] de Falco M, Penta R, Laforgia V, Cobellis L, De Luca A (2005). Apoptosis and human placenta: expression of proteins belonging to different apoptotic pathways during pregnancy. *Journal of Experimental & Clinical Cancer Research*.

[8] Yin Y, Huang W, Lin C, Chen H, MacKenzie A, Ma L (2008). Estrogen suppresses uterine epithelial apoptosis by inducing Birc1 expression. *Molecular Endocrinology*.

[9] Kayisli UA, Selam B, Guzeloglu-Kayisli O, Demir R, Arici A (2003). Human chorionic gonadotropin contributes to maternal immunotolerance and endometrial apoptosis by regulating fas-fas ligand system. *Journal of Immunology*.

[10] Kolialexi A, Tsangaris GT, Antsaklis A (2001). Apoptosis in maternal peripheral blood during pregnancy. *Fetal Diagnosis and Therapy*.

[11] von Dadelszen P, Watson RWG, Noorwali F (1999). Maternal neutrophil apoptosis in normal pregnancy, preeclampsia, and normotensive intrauterine growth restriction. *American Journal of Obstetrics and Gynecology*.

[12] von Rango U, Krusche CA, Kertschanska S, Alfer J, Kaufmann P, Beier HM (2003). Apoptosis of extravillous trophoblast cells limits the trophoblast invasion in uterine but not in tubal pregnancy during first trimester. *Placenta*.

[13] Gleicher N, Vidali A, Karande V (2002). The immunological “wars of the roses”: disagreements amongst reproductive immunologists. *Human Reproduction*.

[14] Huppertz B, Frank H-G, Kingdom JCP, Reister F, Kaufmann P (1998). Villous cytotrophoblast regulation of the syncytial apoptotic cascade in the human placenta. *Histochemistry and Cell Biology*.

[15] von Rango U (2008). Fetal tolerance in human pregnancy—a crucial balance between acceptance and limitation of trophoblast invasion. *Immunology Letters*.

[16] Janeway CA, Travers P, Walport M, Shlomchik KJ (2005). *Immune Biology: The Immune System in Health and Disease*.

[17] Fallarino F, Grohmann U, Hwang KW (2003). Modulation of tryptophan catabolism by regulatory T cells. *Nature Immunology*.

[18] Sabapatha A, Gercel-taylor C, Taylor DD (2006). Specific isolation of placenta-derived exosomes from the circulation of pregnant women and their immunoregulatory consequences. *American Journal of Reproductive Immunology*.

[19] Kvirkvelia N, Vojnovic I, Warner TD (2002). Placentally derived prostaglandin E2 acts via the EP4 receptor to inhibit IL-2-dependent proliferation of CTLL-2 t cells. *Clinical and Experimental Immunology*.

[20] Volumenie JL, Mogneti B, de Smedt D, Menu E, Chaouat G (1997). Induction of transient murine T cell anergy by a low molecular weight compound obtained from supernatants of human placental cultures is linked to defective phosphorylation of TCR CD3 chain. *The American Journal of Reproductive Immunology*.

[21] Lee FW, Boomer JS, Gilman-sachs A (2001). Regeneration and tolerance factor of the human placenta induces IL-10 production. *European Journal of Immunology*.

[22] Boomer JS, Lee GW, Givens TS, Gilman-Sachs A, Beaman KD (2000). Regeneration and tolerance factor's potential role in T-cell activation and apoptosis. *Human Immunology*.

[23] Boomer JS, Derks RA, Lee GW, DuChateau BK, Gilman-Sachs A, Beaman KD (2001). Regeneration and tolerance factor is expressed during T-lymphocyte activation and plays a role in apoptosis. *Human Immunology*.

[24] Fournel S, Aguerre-Girr M, Huc X (2000). Cutting edge: soluble HLA-G1 triggers CD95/CD95 ligand-mediated apoptosis in activated CD8+ cells by interacting with CD8. *Journal of Immunology*.

[25] Hunt JS, Langat DL (2009). HLA-G: a human pregnancy-related immunomodulator. *Current Opinion in Pharmacology*.

[26] Vacchio MS, Hodes RJ (2005). Fetal expression of fas ligand is necessary and sufficient for induction of CD8 T cell tolerance to the fetal antigen H-Y during pregnancy. *The Journal of Immunology*.

[27] Priddy KD (1997). Immunologic adaptations during pregnancy. *Journal of Obstetric, Gynecologic, and Neonatal Nursing*.

[28] Weetman AP (1999). The immunology of pregnancy. *Thyroid*.

[29] Shinoda R, Watanabe M, Nakamura Y, Maruoka H, Kimura Y, Iwatani Y (2003). Physiological changes of Fas expression in peripheral lymphocyte subsets during the menstrual cycle. *Journal of Reproductive Immunology*.

[30] Reinhard G, Noll A, Schlebusch H, Mallmann P, Ruecker AV (1998). Shifts in the TH1/TH2 balance during human pregnancy correlate with apoptotic changes. *Biochemical and Biophysical Research Communications*.

[31] Shirshev SV, Kuklina EM, Yarilin AA (2003). Role of reproductive hormones in control of apoptosis of T-lymphocytes. *Biochemistry*.

[32] Hofmann-Lehmann R, Holznagel E, Lutz H (1998). Female cats have lower rates of apoptosis in peripheral blood lymphocytes than male cats: correlation with estradiol-17*β*, but not with progesterone blood levels. *Veterinary Immunology and Immunopathology*.

[33] Scudeletti M, Lanza L, Monaco E (1999). Immune regulatory properties of corticosteroids: prednisone induces apoptosis of human T lymphocytes following the CD3 down-regulation. *Annals of the New York Academy of Sciences*.

